# Antifungal Susceptibilities of Bloodstream Isolates of *Candida* Species from Nine Hospitals in Korea: Application of New Antifungal Breakpoints and Relationship to Antifungal Usage

**DOI:** 10.1371/journal.pone.0118770

**Published:** 2015-02-23

**Authors:** Eun Jeong Won, Jong Hee Shin, Min Ji Choi, Wee Gyo Lee, Yeon-Joon Park, Young Uh, Shine-Young Kim, Mi-Kyung Lee, Soo Hyun Kim, Myung Geun Shin, Soon Pal Suh, Dong Wook Ryang

**Affiliations:** 1 Department of Laboratory Medicine, Chonnam National University Medical School, Gwangju, Republic of Korea; 2 Department of Laboratory Medicine, Ajou University School of Medicine, Suwon, Republic of Korea; 3 Department of Laboratory Medicine, The Catholic University of Korea, College of Medicine, Seoul, Republic of Korea; 4 Department of Laboratory Medicine, Yonsei University Wonju College of Medicine, Wonju, Republic of Korea; 5 Department of Laboratory Medicine, Pusan National University Hospital, Busan, Republic of Korea; 6 Department of Laboratory Medicine, Chung-Ang University College of Medicine, Seoul, Republic of Korea; California Department of Public Health, UNITED STATES

## Abstract

We applied the new clinical breakpoints (CBPs) of the Clinical and Laboratory Standards Institute (CLSI) to a multicenter study to determine the antifungal susceptibility of bloodstream infection (BSI) isolates of *Candida* species in Korea, and determined the relationship between the frequency of antifungal-resistant *Candida* BSI isolates and antifungal use at hospitals. Four hundred and fifty BSI isolates of *Candida* species were collected over a 1-year period in 2011 from nine hospitals. The susceptibilities of the isolates to four antifungal agents were determined using the CLSI M27 broth microdilution method. By applying the species-specific CBPs, non-susceptibility to fluconazole was found in 16.4% (70/428) of isolates, comprising 2.6% resistant and 13.8% susceptible-dose dependent isolates. However, non-susceptibility to voriconazole, caspofungin, or micafungin was found in 0% (0/370), 0% (0/437), or 0.5% (2/437) of the *Candida* BSI isolates, respectively. Of the 450 isolates, 72 (16.0%) showed decreased susceptibility to fluconazole [minimum inhibitory concentration (MIC) ≥4 μg/ml]. The total usage of systemic antifungals varied considerably among the hospitals, ranging from 190.0 to 7.7 defined daily dose per 1,000 patient days, and fluconazole was the most commonly prescribed agent (46.3%). By Spearman’s correlation analysis, fluconazole usage did not show a significant correlation with the percentage of fluconazole resistant isolates at hospitals. However, fluconazole usage was significantly correlated with the percentage of fluconazole non-susceptible isolates (r = 0.733; P = 0.025) or the percentage of isolates with decreased susceptibility to fluconazole (MIC ≥4 μg/ml) (r = 0.700; P = 0.036) at hospitals. Our work represents the first South Korean multicenter study demonstrating an association between antifungal use and antifungal resistance among BSI isolates of *Candida* at hospitals using the new CBPs of the CLSI.

## Introduction

The incidence of *Candida* bloodstream infections (BSIs) has increased over the past several decades, and antifungal use has increased drastically worldwide [1, 2]. Although *Candida albicans* remains the leading *Candida* species that causes BSI, a shift in the epidemiology of *Candida* BSI toward greater isolation of non-*albicans Candida* species has been a global concern in the past two decades [1, 3]. Several studies have evaluated the relationship between antifungal drug use and changes in the epidemiology of candidemia [4–6]; however, to date, few multicenter surveillance studies have been conducted on the relationship between antifungal drug use and the frequency of antifungal resistance of *Candida* BSI isolates at hospitals. Moreover, several surveillance programs on *Candida* isolates responsible for BSI worldwide have not reported alterations in the patterns of susceptibility to the azoles or echinocandins over time [1, 3, 5, 6]. The cause may be the rarity of acquired antifungal resistance among BSI *Candida* isolates or due to the antifungal susceptibility tests and breakpoints being non-optimal for the detection of resistance in *Candida* isolates [7].

The Clinical and Laboratory Standards Institute (CLSI) recently developed new *Candida* species-specific clinical breakpoints (CBPs) for fluconazole, voriconazole, and echinicandins [7, 8]. A recent report has shown that resistance to the azoles and echinocandins of *Candida* species may be increased using the new CLSI CBPs [9]. Therefore, the new CBPs of the CLSI may be applied to antifungal surveillance studies as sensitive tools for detecting emerging resistance in *Candida* BSI isolates. In the present study, we applied new species-specific CLSI CBPs in a multicenter study to determine the antifungal susceptibility of BSI isolates of *Candida* species in Korea, and investigated the relationship between the frequency of antifungal resistance of *Candida* BSI isolates and antifungal use at nine hospitals.

## Materials and Methods

A surveillance study was conducted at nine university hospitals (A-I) located throughout Korea to determine the frequency of antifungal resistance of *Candida* BSI isolates, and to determine their relationship with antifungal use. The participant hospitals were as follows: Ajou University School of Medicine, Suwon; The Catholic University College of Medicine, Seoul; Chonnam National University Hwasun Hospital, Hwasun; Chonnam National University Hospital, Gwangju; Chonbuk National University of Medicine, Jeonju; Chung-Ang University College of Medicine, Seoul; Pusan National University Yangsan Hospital, Yangsan; Yonsei University College of Medicine, Seoul; and Yonsei University Wonju College of Medicine, Wonju. Among the nine hospitals, six had more than 1,000 beds, while the other three (B, D, and I) had 500–1,000 beds. During the 1-year period from January to December 2011, each participant hospital was required to collect blood isolates (one isolate per patient) of *Candida* species. The annual usage of systemic antifungal agents for patients admitted to each hospital between January and December 2011 was determined by calculating the number of defined daily doses per 1,000 patient days (DDD/1,000 PD), as specified by the WHO ATC/DDD system (www.whocc.no/atcddd/) and the DDD measurement methodology [10]. In addition, the incidence of candidemia was defined as the number of cases of candidemia per 10,000 patient days (PD).

Species identification and antifungal susceptibility testing were performed at Chonnam National University Hospital for all 450 isolates. This study was approved by the institutional review board of Chonnam National University Hospital (IRB CNUH-2011-026). A waiver of consent was granted given the observational nature of the project. The study involved only the results of the species identification and antifungal susceptibility testing of *Candida* species isolated from routine cultures in the mycology laboratory, and no information was used that could lead to patient identification. Species identification was based on colony morphology using CHROMagar *Candida* and a commercial system (API 20C; bioMérieux, Marcy L’Étoile, France) (Vitek 2 system; Vitek 2 YST; bioMérieux) or sequencing [11]. Susceptibility to fluconazole, voriconazole, caspofungin, and micafungin was assessed by the CLSI BMD method M27-A3 after 24 h and using the new CLSI-developed CBPs [8, 12]. Revised CLSI CBPs were applied to 437 isolates of six *Candida* species, including *Candida albicans*, *Candida parapsilosis*, *Candida tropicalis*, *Candida glabrata*, *Candida guilliermondii* and *Candida krusei*, for both caspofungin, and micafungin, while the revised CLSI CBPs were applied to 370 isolates of four common species (*C*. *albicans*, *C*. *parapsilosis*, *C*. *tropicalis*, and *C*. *krusei*) for voriconazole. Revised CLSI CBPs for fluconazole were applied to four common *Candida* isolates (*C*. *albicans*, *C*. *parapsilosis*, *C*. *tropicalis*, and *C*. *glabrata*), and all *C*. *krusei* isolates are considered resistant to fluconazole irrespective of the minimum inhibitory concentration (MIC) [8]. Two reference strains, *C*. *parapsilosis* ATCC 22019 and *C*. *krusei* ATCC 6258, were included in each test as quality control isolates. The relationship between antifungal (total or individual) usage and the incidence of candidemia or antifungal susceptibility was determined using Spearman’s rank correlation coefficient (rho, *r*) and its corresponding *P* value. A *P* value less than 0.05 was deemed to indicate significance in both analyses.

## Results and Discussion

Table 1 summarizes the *in vitro* susceptibility of 450 *Candida* isolates to azoles (fluconazole, voriconazole) and echinocandins (caspofungin and micafungin). Of the 450 total BSI isolates from nine hospitals, 16.0% (72/450) had fluconazole MICs ≥4 μg/ml. By applying the species-specific new CBPs, resistance and susceptible-dose dependence to fluconazole were found in 2.6% (11/428) and 13.8% (59/428) of four common *Candida* species and *C*. *krusei*, respectively. However, no voriconazole, caspofungin or micafungin resistance was detected in any species, with the exception of only two *C*. *parapsilosis* isolates which exhibited intermediate resistance (MIC, 4 μg/ml) to micafungin. Overall, *Candida* BSI isolates remained largely susceptible to the three antifungals, with susceptibility rates of 100%, 100%, and 99.5% for voriconazole caspofungin, and micafungin, respectively. These findings indicate that the current rate of resistance to voriconazole and two echinocandins among *Candida* species is low in Korea; this trend is similar to our previous report [13]. However, susceptibility to fluconazole was found in 83.6% (358/428) of the isolates by the new CBPs, which is significantly lower than our previous report (96.4% susceptible, *P* <0.005) based on the original CBPs [13].

**Table 1:** Susceptibility to azoles and echinocandins of 450 *Candida* bloodstream isolates from nine hospitals as determined by the CLSI method.

Species (No. of isolates)	Antifungal agent	MIC ranges (μg/ml)	No. (%) of isolates by new CBPs ^a^				No. (%) of isolates with MIC ≥4 μg/ml
			Susceptible	SDD / I	Resistance	NA	
*C*. *albicans* (183)	Fluconazole	0.125–4	182	1	0	0	1
	Voriconazole	0.03–0.06	183	0	0	0	0
	Caspofungin	0.003–0.125	183	0	0	0	0
	Micafungin	0.003–0.03	183	0	0	0	0
*C*. *parapsilosis* (101)	Fluconazole	0.25–8	96	4	1	0	5
	Voriconazole	0.03–0.125	101	0	0	0	0
	Caspofungin	0.06–1	101	0	0	0	0
	Micafungin	0.125–4	99	2	0	0	2
*C*. *tropicalis* (82)	Fluconazole	0.125–4	80	2	0	0	2
	Voriconazole	0.03–0.125	82	0	0	0	0
	Caspofungin	0.003–0.25	82	0	0	0	0
	Micafungin	0.003–0.125	82	0	0	0	0
*C*. *glabrata* (58)	Fluconazole	1- >64	0	52	6	0	50
	Voriconazole	0.03–2	NA	NA	NA	58	0
	Caspofungin	0.003–0.125	58	0	0	0	0
	Micafungin	0.015–0.06	58	0	0	0	0
*C*. *guilliermondii* (9)	Fluconazole	1–8	NA	NA	NA	9	5
	Voriconazole	0.03–0.125	NA	NA	NA	9	0
	Caspofungin	0.125–1	9	0	0	0	0
	Micafungin	0.06–2	9	0	0	0	0
*C*. *krusei* (4) ^b^	Fluconazole	16–16	0	0	4	0	4
	Voriconazole	0.125–0.25	4	0	0	0	0
	Caspofungin	0.06–0.25	4	0	0	0	0
	Micafungin	0.03–0.25	4	0	0	0	0
Others (13) ^c^	Fluconazole	0.25–8	NA	NA	NA	13	5
	Voriconazole	0.03–0.125	NA	NA	NA	13	0
	Caspofungin	0.06–0.125	NA	NA	NA	13	0
	Micafungin	0.012–0.25	NA	NA	NA	13	0
Total (450)	Fluconazole	0.125- >64	358 (83.6)	59 (13.8)	11 (2.6)	22	72 (16.0)
	Voriconazole	0.03–2	370 (100)	0 (0)	0 (0)	80	0 (0)
	Caspofungin	0.003–1	437 (100)	0 (0)	0 (0)	13	0 (0)
	Micafungin	0.006–4	435 (99.5)	2 (0.5)	0 (0)	13	2 (0.4)

^a^ CBP, clinical breakpoints by the CLSI were obtained from references [7, 8]; R and SDD, resistant and susceptible-dose dependent; NA, non-applicable because species-specific CBPs are not at present available by the CLSI.

^b^ Isolates of *C*. *krusei* are considered resistant to fluconazole, irrespective of the MIC.

^c^ Others includes *Candida pelliculosa* (5 isolates), *Candida lusitaniae* (2 isolates), *Candida intermedia* (2 isolates), *Candida haemulonii* (1 isolate), *Candida lipolytica* (1 isolate), *Candida melibiosica* (1 isolate) and *Candida orthopsilosis* (1 isolate).

Several global surveillance programs have demonstrated that most BSIs caused by three common *Candida* species (i.e., *C*. *albicans*, *C*. *tropicalis*, and *C*. *parapsilosis*) are susceptible to fluconazole using the original CLSI CBPs [3, 13]. During the past few years, the CLSI has adjusted CBPs for fluconazole by lowering them to the same MIC values (S, ≤ 2 μg/mL; susceptible dose dependent [SDD], 4 μg/mL; R, ≥ 8 μg/mL) as the European Committee on Antimicrobial Susceptibility Testing (EUCAST) for three common *Candida* species [14]. Using these species-specific CBPs, trends toward increased resistance to fluconazole in these three species were noted [9, 15]. Similarly, 2.2% (8/366) of isolates of three common *Candida* species in the present study were categorized as fluconazole non-susceptible (resistant or SDD) based on the revised CLSI CBPs, while none were categorized as fluconazole non-susceptible based on the original CBPs (fluconazole MIC, ≥16 μg/ml). Additionally, only 34.5% (20/58) of *C*. *glabrata* were categorized as fluconazole non-susceptible (10.3% resistant or 24.1% SDD) based on the original CBPs, but all 58 isolates of *C*. *glabrata* were categorized as fluconazole non-susceptible (10.3% resistant and 89.7% SDD), suggesting that all *C*. *glabrata* isolates were no longer considered susceptible to fluconazole using revised CBPs [7, 8, 14]. In 2013, the EUCAST also defined fluconazole CBPs for *C*. *glabrata* (intermediate, 0.002 to 32 μg/ml; resistant, >32 μg/ml; [http://www.eucast.org/clinical_breakpoints/; version 7.0]), suggesting that a harmonization of CBPs between the CLSI and the EUCAST for fluconazole has been achieved for the four most common *Candida* species [14, 16]. Overall, these data show that non-susceptibility to fluconazole of *Candida* species may be increased using the new CLSI CBPs, which may thus be a sensitive measure for detecting the emergence of *Candida* strains with decreased fluconazole susceptibility, a finding that is consistent with other reports [7, 9].

The incidence of candidemia, antifungal agent consumption, and the percentage of fluconazole non-susceptible *Candida* BSI isolates at nine Korean hospitals are shown in Table 2. The average incidence of candidemia at the nine university hospitals was 2.01 (1.14 to 2.70) per 10,000 PD. The most prevalent species was *C*. *albicans* (average: 0.79 episodes/10,000 PD; 40.4%) followed by *C*. *parapsilosis* (19.9%), *C*. *tropicalis* (17.6%), and *C*. *glabrata* (14.2%). There was considerable variation in total antifungal use at the nine hospitals, ranging from 7.7 to 190.0 DDD/1,000 PD. Fluconazole was the most common agent (average: 29.3 DDD/1,000 PD; 46.3%), followed by itraconazole (20.5%), amphotericin B (13.6%), voriconazole (7.4%), lipid formulation of amphotericin B (7.3%), and the echinocandins (4.9%). Oral fluconazole represented 77.5% of the total fluconazole usage at the nine hospitals (36.0% of the total antifungal usage). The mean total usage of antifungals was 63.2 DDD/PD, which was higher than that in 2005 (39.2 DDD/PD) [17]. In addition, the mean usage of fluconazole more than doubled from 2005 (11.7 DDD/1,000PD) to 2011 (29.3 DDD/1,000 PD) [17]. Accordingly, the rate of fluconazole resistance (MIC ≥64 μg/ml) of *C*. *glabrata* was increased to 10.3% (6/58) in the present study (2011), compared with 6% (2/72) in our previous multicenter study (2006–2007) [13].

**Table 2:** The incidence of candidemia, antifungal drug usage, and fluconazole susceptibility among bloodstream isolates of *Candida* species at nine university hospitals in 2011.

Parameters			Hospital (Annual patient-days)									Average (%)
			A	B	C	D	E	F	G	H	I	
			(412,959)	(220,761)	(361,580)	(219,562)	(683,220)	(329,957)	(387,335)	(364,687)	(266,017)	
Incidence of candidemia episodes/10,000 patient-days												
	Three common species											
		*C*. *albicans*	0.48	0.59	0.91	1.18	1.23	1.03	0.34	0.85	0.53	0.79 (40.4)
		*C*. *parapsilosis*	0.31	0.45	0.5	0.55	0.31	0.88	0.26	0.47	0.08	0.42 (19.9)
		*C*. *tropicalis*	0.22	0.27	0.3	0.18	0.48	0.45	0.34	0.44	0.38	0.34 (17.6)
		Subtotal	1.02	1.31	1.71	1.91	2.02	2.36	0.93	1.75	0.98	1.6 (78.0)
	Other than three common species											
		*C*. *glabrata* ^b^	0.41	0.18	0.41	0.5	0.38	0.15	0.15	0.11	0.23	0.28 (14.2)
		*C*. *guilliermondii*	0	0.05	0.08	0.05	0.03	0.15	0	0.05	0	0.05 (2.1)
		Others	0.07	0.18	0.28	0.23	0.09	0.03	0.05	0.16	0.04	0.13 (5.7)
		Subtotal ^b^ ^,^ ^c^	0.48	0.41	0.77	0.77	0.5	0.33	0.21	0.33	0.26	0.45 (22.0)
	Total, all *Candida* species		1.5	1.72	2.49	2.69	2.52	2.7	1.14	2.08	1.24	2.01 (100)
Usage of antifungal drug, defined daily dose/1,000 patient-days												
	Fluconazole, total		59.8	58.4	43.7	43.2	28.4	14	7.5	4.5	4.1	29.3 (46.3)
		Oral	56.6	32.1	35.5	38.3	23.6	8.6	7.5	0.5	1.8	22.7 (36.0)
		Intravenous	3.3	26.3	8.2	4.9	4.8	5.4	0.0	4.0	2.3	6.6 (10.4)
	Itraconazole		15.7	68.8	5.3	4.3	5.5	2.1	1.9	1.6	11.5	13 (20.5)
	Amphotericin B		18.9	15.6	5.7	4.7	20.5	2.5	6.8	0	2.8	8.6 (13.6)
	Voriconazole		12.7	16.9	2.1	1.2	0.8	5.6	1.4	0.4	0.9	4.6 (7.4)
	Lipid formulation of amphotericin B		16	21.6	2.3	0	0	0	0.4	0.7	0.6	4.6 (7.3)
	Caspofungin		5.5	5.2	0.8	0	0.6	0.5	0.4	0.4	0.7	1.6 (2.5)
	Micafungin		4.9	3.4	0.7	0.6	1.9	0	0.7	0.1	1.2	1.5 (2.4)
	Anidulafungin		0	0.1	0	0	0	0	0	0.1	0	0 (0.0)
	Total		133.4	190	60.6	54	57.7	24.7	19.1	7.7	21.9	63.2 (100)
The percentage of isolates (No. with indicated result / total no. tested) by the CLSI-M27A^a^												
	Fluconazole resistant		12.5 (6/48)	10.0 (2/20)	0.0 (0/35)	0.0 (0/40)	1.1 (1/94)	0.0 (0/77)	0.0 (0/37)	3.4 (2/58)	0.0 (0/19)	2.6 (11/428)
	Fluconazole non-susceptible ^b^ ^,^ ^c^		33.3 (16/48)	15.0 (3/20)	22.9 (8/35)	20.0 (8/40)	17.0 (16/94)	7.8 (6/77)	18.9 (7/37)	8.6 (5/58)	5.3 (1/19)	16.4 (70/428)
	Fluconazole MIC ≥4 μg/ml ^c^ ^,^ ^d^		26.5 (13/49)	14.3 (3/21)	22.2 (8/36)	13.3 (6/45)	16.0 (15/94)	11.9 (10/84)	20.5 (8/39)	12.7 (8/63)	5.3 (1/19)	16.0 (72/450)

^a^ The percentage of fluconazole resistant or non-susceptible (resistant or susceptible dose-dependent) isolates by revised CLSI CBPs. Revised CLSI CBPs for fluconazole were applied to four common *Candida* isolates (*C*. *albicans*, *C*. *parapsilosis*, *C*. *tropicalis*, and *C*. *glabrata*), and all *C*. *krusei* isolates were considered resistant to fluconazole irrespective of the MIC [8].

^b^ *P* < 0.05, significant relationship between oral fluconazole usage and a given category by Spearman correlation analysis.

^c^ *P* < 0.05, significant relationship between total fluconazole usage and a given category by Spearman correlation analysis.

^d^ The percentage of isolates with decreased susceptibility to fluconazole (MIC ≥4 μg/ml) in all bloodstream isolates tested.

The use of fluconazole has been suggested to be associated with an increased risk of candidemia caused by non-*albicans Candida* species [4, 18]. However, serial follow-up studies demonstrated that antifungal usage increased significantly, but the incidence of non-*albicans* candidemia was maintained [5, 6]. In the present study, no relationship was found between antifungal use and the incidence of candidemia caused by all non-*albicans Candida* or all *Candida* species. However, the incidence of candidemia caused by all *Candida* species other than three common species showed positive correlations with usage of total (oral and intravenous) fluconazole (*r* = 0.681, *P* = 0.044) or oral fluconazole (*r* = 0.773, *P* = 0.015). In addition, oral fluconazole usage showed a significant positive correlation with the incidence of candidemia caused by *C*. *glabrata* (*r* = 0.832, *P* = 0.005). This is supported by the report that the most of *C*. *albicans*, *C*. *tropicalis*, and *C*. *parapsilosis* are highly susceptible to fluconazole, but many of the less common *Candida* species, including *C*. *glabrata*, exhibit decreased susceptibility to fluconazole [19].

**Fig 1:**
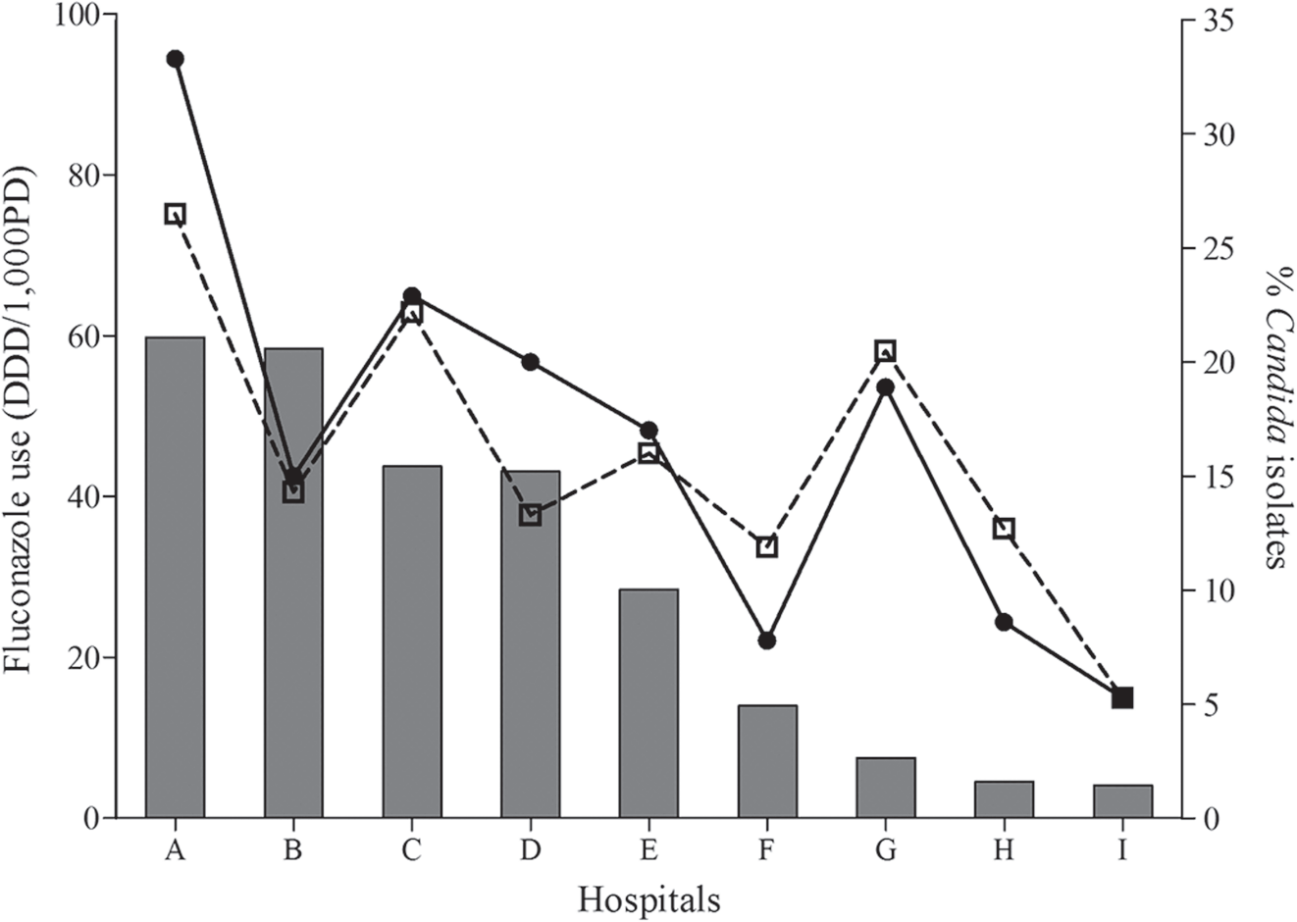
Relationship between the fluconazole usage and the percentage of isolates with non-susceptible to fluconazole, or isolates with decreased susceptibility to fluconazole (MIC ≥4 μg/ml) at nine university hospitals in Korea. The usage of fluconazole, defined as the daily dose/1,000 patient days (DDD/1,000 PD) at the individual hospital was represented by the grey columns. The percentage of isolates with non-susceptible to fluconazole (closed circle with solid line) and the percentage of isolates with decreased susceptibility to fluconazole (MIC ≥4 μg/ml) (open rectangle with dotted line) showed positive correlations with the usage of fluconazole at the individual hospitals.

When the species-specific new CBPs were applied, the percentage of fluconazole non-susceptible *Candida* BSI isolates varied among the nine hospitals from 5.3% to 33.3% (Table 2). Because revised species-specific CBPs are not at present available for less common *Candida* species, we also assessed the percentage of decreased susceptibility to fluconazole (MIC ≥4 μg/ml) in all 450 BSI isolates, which also varied among the hospitals from 5.3% to 26.5%. By Spearman’s correlation analysis, total fluconazole usage showed positive correlations with the percentage of isolates with decreased susceptibility to fluconazole (MIC ≥4 μg/ml) (*r* = 0.700, *P* = 0.036) at hospitals (Fig. 1). Moreover, the incidence of candidemia caused by fluconazole non-susceptible *Candida* isolates showed positive correlations with usage of total fluconazole (*r* = 0.733, *P* = 0.025) or oral fluconazole (*r* = 0.8, *P* = 0.01).

In the present study, we evaluated the annual usage of systemic antifungal agents for patients admitted to each hospital. However, given the fact that many of the hematologic patients are exposed to heavy antifungal use as outpatients, oral fluconazole use for outpatients also may have an influence on the frequency of antifungal-resistant *Candida* BSI isolates at hospitals. Among nine hospitals, the higher use of antifungal agents including fluconazole was found in two hospitals (hospitals A and B) which reported a higher population of immunocompromised patients (mainly hematological malignancies). The percentages of fluconazole resistant isolates, as expected, were higher in these two hospitals than those in other hospitals (11.8% vs. 0.8%, *P* = 0.001). However, Spearman’s analysis indicated no significant relationship was found between antifungal usage (total or individual) and the presence of fluconazole-resistant *Candida* species among nine hospitals.

Recent two studies have shown that patients with candidemia due to fluconazole non-susceptible *Candida* species are more likely to have received prior or recent fluconazole therapy [20, 21]. In addition, suboptimal initial dosing of prior fluconazole therapy is associated with candidemia with fluconazole-non susceptible *Candida* species [21]. Although the present study found an association, not causality, we first describe here that hospitals with a higher rate of fluconazole use exhibited a higher incidence of candidemia due to fluconazole non-susceptible *Candida* isolates, using revised CLSI CBPs. We also add evidence on a significant relationship between fluconazole use and the incidence of candidemia caused by all *Candida* species other than three common species. To our knowledge, this work represents the first multicenter study to demonstrate an association between antifungal use and the frequency of antifungal resistance of *Candida* BSI isolates at hospitals.

Changing trends in the epidemiology of candidemia and the emergence of antifungal resistance have affected selection of appropriate antifungal agents [1, 7]. A recent study in a hospital showed that an increase in the use of echinocandins was associated with a decrease in the incidence of *C*. *parapsilosis* or *C*. *guilliermondii* candidemia and an increase in the incidence of *C*. *tropicalis* candidemia [4]. However, our data suggested no relationship between the usage of echinocandins and the incidence of candidemia. This might be due to the limited use of echinocandins in Korean hospitals. At present, fluconazole as a commonly used antifungal agent is suggested to contribute to the changing epidemiology of candidemia at Korean hospitals. However, given the increasing use of antifungal drugs, including echinocandins, continuous national surveillance programs using the new antifungal CBPs are needed to identify changes in the antifungal susceptibility patterns of *Candida* BSI isolates.
